# Supplementary material for: A human stem cell resource to decipher the biochemical and cellular basis of neurodevelopmental defects in Lowe syndrome
Source: Biol Open. 2022 Feb 4;11(1):bio059066. doi: 10.1242/bio.059066 (PMC8822356; doi:10.1242/bio.059066)
Supplement: Supplementary information [file biolopen-11-059066-s1.pdf]

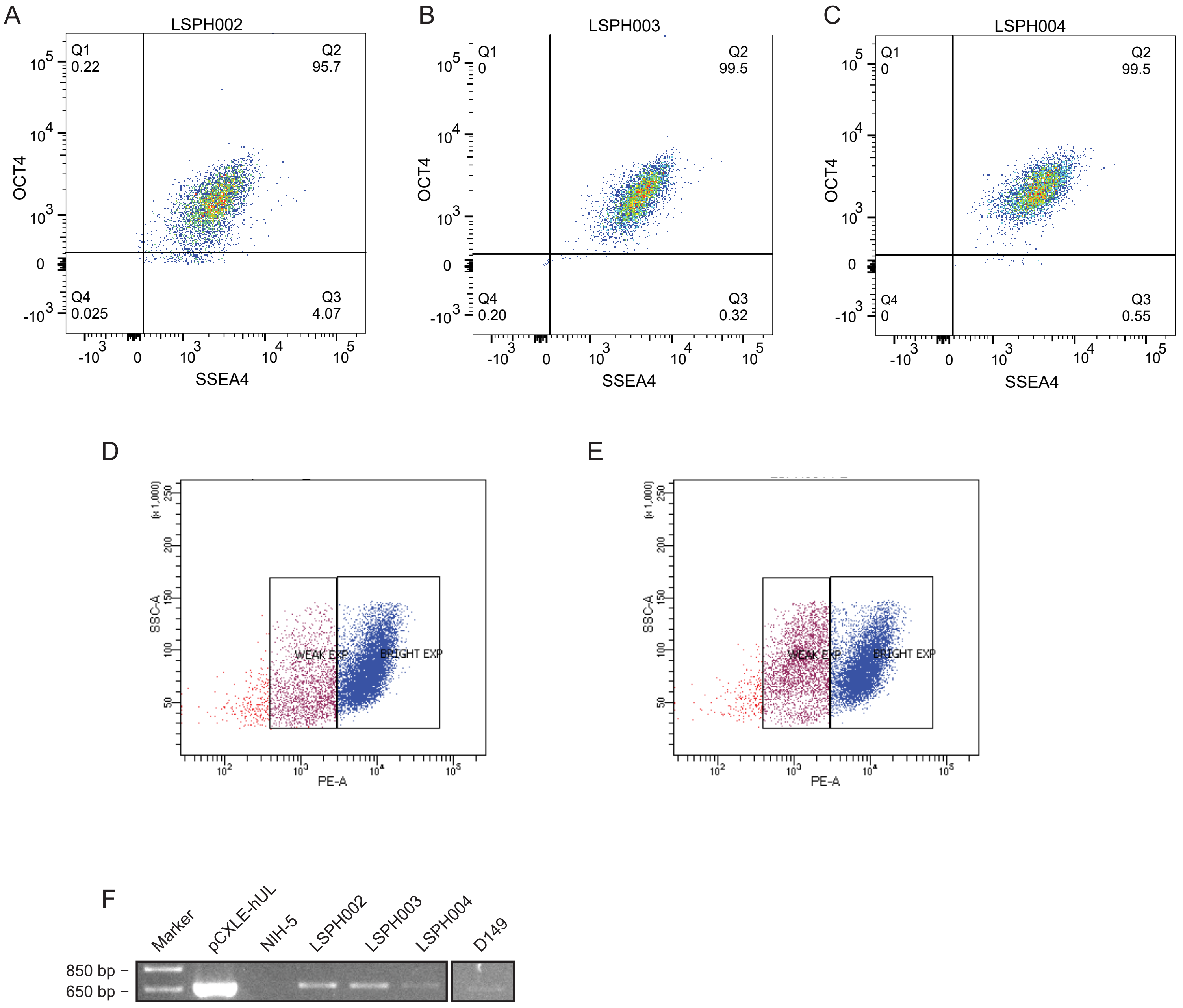

Fig. S1. (A-C) Presence of pluripotency markers OCT4 and SSEA4 in the hiPSC patient lines shown by quantitative FACS analysis. (D-E) Representative flow cytometry data to illustrate fluorescence activated cell sorting of NSCs using CD133-PE conjugated primary antibody. (F) PCR analysis of EBNA1 to assess plasmid footprint in feeder-free iPSC lines LSPH002, LSPH003 and LSPH004. Positive control - Plasmid pCXLE-hUL; Negative control - iPSC NIH5.

Biology Open • Supplementary information

Biology Open (2022): doi:10.1242/bio.059066: Supplementary information

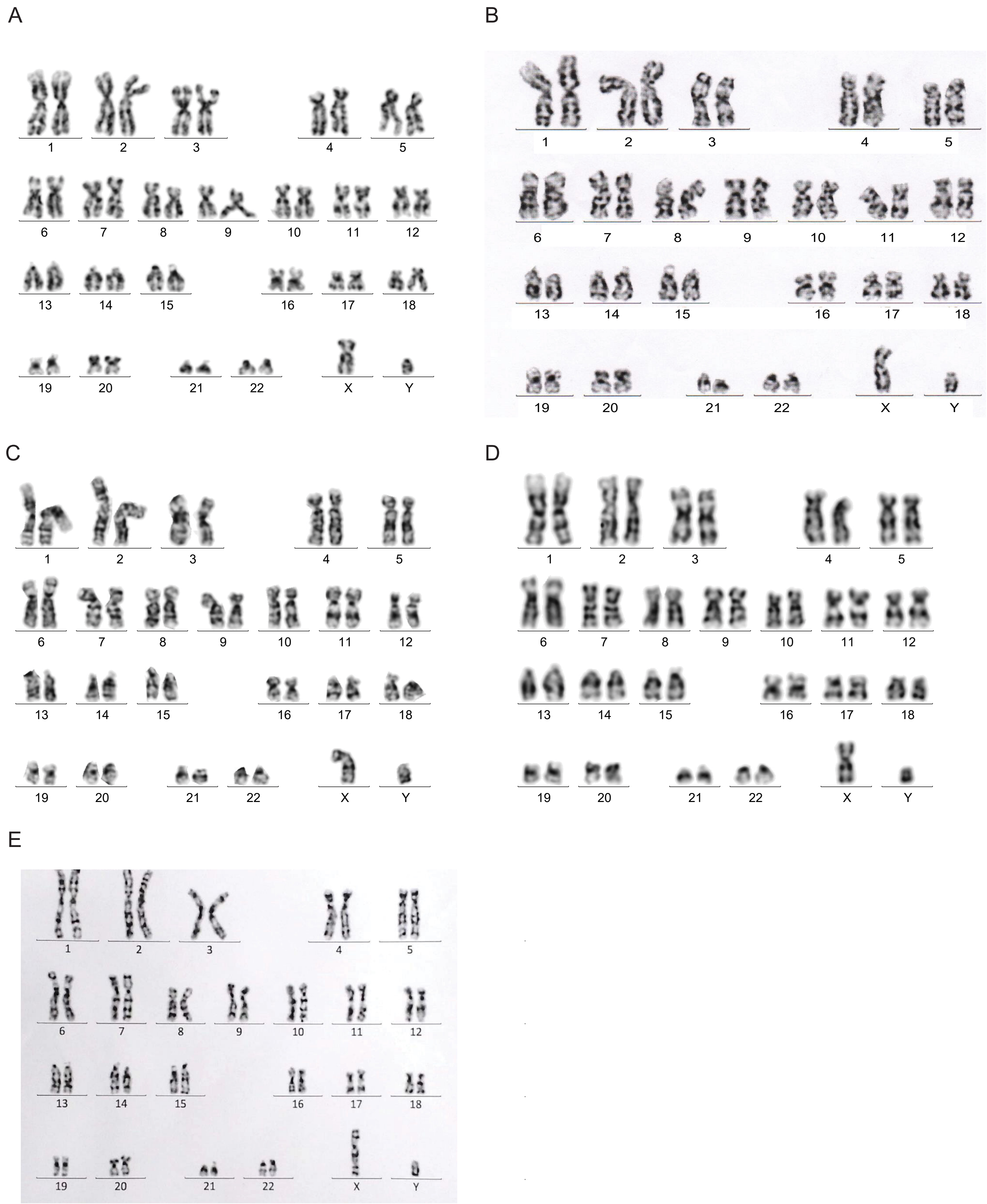

Fig. S2. Karyogram depicting the karyotype 46(X,Y) of (A) LCL LSPH002 (B) LCL LSPH003 (C) LCL LSPH004 (D) hiPSC LSPH002 (E) hiPSC LSPH003.

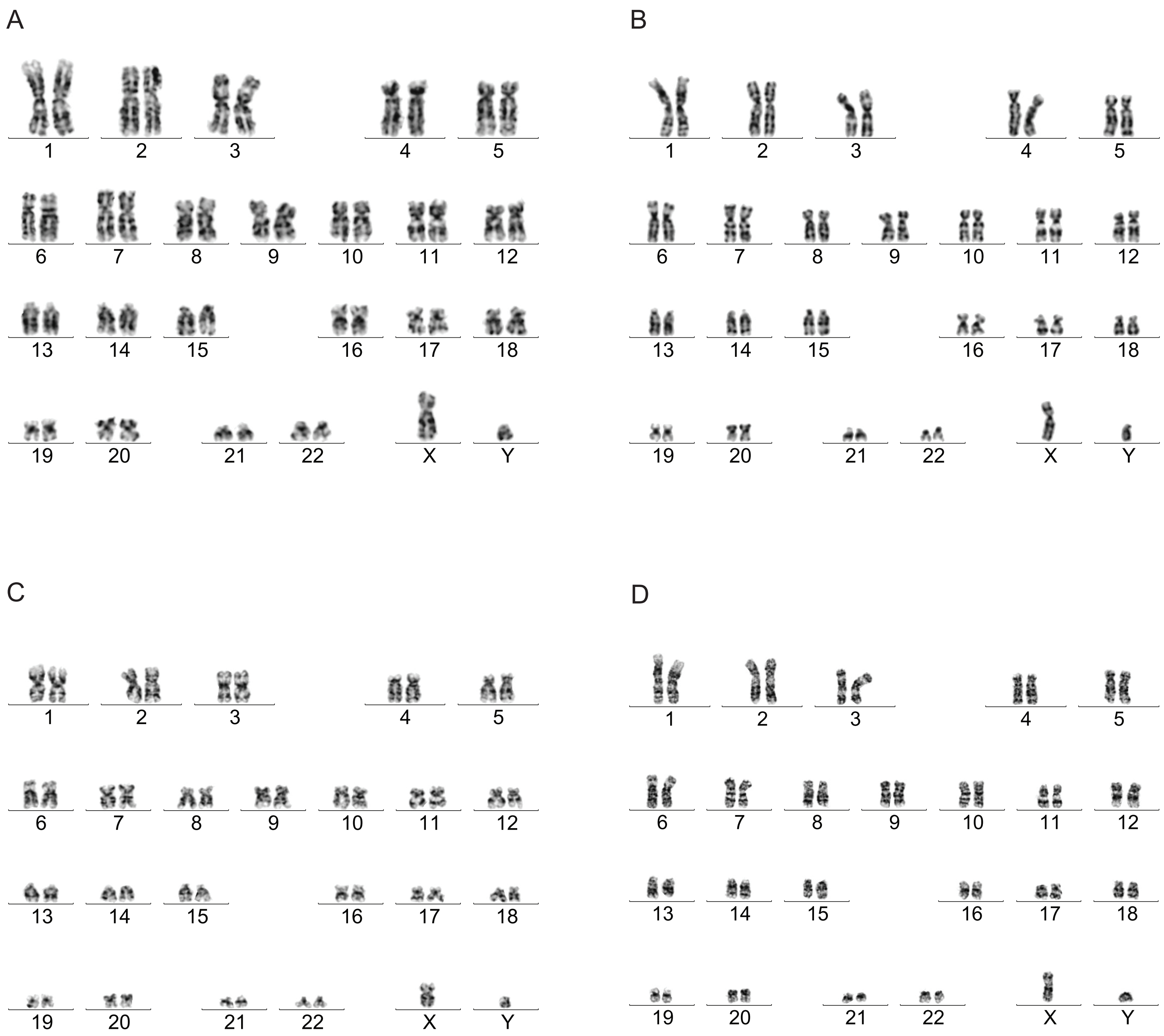

Fig. S3. Karyogram depicting the karyotype 46(X,Y) of (A) NSC D149 (B) NSC LSPH004 (C) NSC D149-GSH (D) NSC LSPH004-GSH.

Biology Open • Supplementary information

Table S1. Short tandem repeat analysis of blood, LCL and iPSC derived DNA for LSPH002, LSP003 and LSPH004. Data for all ten loci assayed for STR is shown.

GP Date	Sample No.	Sample type	AMEL	CSF1PO	D13S317	D16S539	D21S11	D5S818	D7S820	TH01	TPOX	vWA
24.04.2018	LSPH002	Blood	X, Y	11,12	12,12	11,12	28,28	12,14	8,11	6,9	11,12	17,18
24.04.2018	LSPH002	LCL	X, Y	11,12	12,12	11,12	28,28	12,14	8,11	6,9	11,12	17,18
24.04.2018	LSPH002	iPSC	X, Y	11,12	12,12	11,12	28,28	12,14	8,11	6,9	11,12	17,18
24.04.2018	LSPH003	Blood	X, Y	11,12	12,12	11,12	28,28	12,14	8,11	6,9	11,12	17,18
24.04.2018	LSPH003	LCL	X, Y	11,12	12,12	11,12	28,28	12,14	8,11	6,9	11,12	17,18
24.04.2018	LSPH003	iPSC	X, Y	11,12	12,12	11,12	28,28	12,14	8,11	6,9	11,12	17,18
24.04.2018	LSPH004	Blood	X, Y	10,13	12,12	12,12	28,30.2	11,12	8,12	8,8	10,11	16,18
24.04.2018	LSPH004	LCL	X, Y	10,13	12,12	12,12	28,30.2	11,12	8,12	8,8	10,11	16,18
24.04.2018	LSPH004	iPSC	X, Y	10,13	12,12	12,12	28,30.2	11,12	8,12	8,8	10,11	16,18

Table S2. Table showing the list of GO categories with upregulated or downregulated genes in RNA derived from LSPH004 iPSC compared to controls. Individual genes included under each GO term is shown in a given row.

Click here to download Table S2

Table S3. Table showing the list of GO categories with upregulated or downregulated genes in RNA derived from LSPH004 NSC compared to controls. Individual genes included under each GO term is shown in a given row.

Click here to download Table S3

Table S4. Table showing the following: Sheet-Source parameters: the mass spectrometer instrument parameters for data acquisition Sheet- iPSC: Multiple reaction monitoring (MRM) parameters for detecting and quantifying PIP, PIP_2 and PE species from iPSC. Sheet- NSC: Multiple reaction monitoring (MRM) parameters for detecting and quantifying PIP, PIP_2 and PE species from NSC.

Click here to download Table S4

Biology Open • Supplementary information

Table S5. List of PCR primers and antibodies in this study is presented in this table. A human stem cell resource to decipher the biochemical and cellular basis of neurodevelopmental defects in Lowe Syndrome**.**

**QRT-PCR primers**		
**Gene name**	**Forward primer (5'-3')**	**Reverse primer (5'-3')**
inositol polyphosphate -5-phosphatase B (INPP5B)	CAAATGGGAAGGGAGTGCCT	ATGAGCCTTGTCCGACACTG
inositol polyphosphate -5-phosphatase D (INPP5D/SHIP1)	GCACCTGGAACATGGGTAAC	TGGGTGCCGATCACGTAAAT
Synaptojanin 1 (SYNJ1)	CCTCACAACCGCAAGTAAAAAC	AGCTGAGCCTTTGATACAGCA
inositol polyphosphate -5-phosphatase J (INPP5J)	CACCCTCCAAGGTGGACTC	CCTTCCGAGGAAGCTGACATA
inositol polyphosphate phosphatase like 1 (INPPL1/SHIP2)	CCAACACCCTGGGGAACAAG	AGTTTTGGTTCCTCCGAGCC
inositol polyphosphate -5-phosphatase F (INPP5F/SAC2)	CTTCCAAGCCAAGGACCACT	AGTAGATCAGTAGCGGGTCG
inositol polyphosphate -5-phosphatase K (INPP5K/SKIP)	ATCCTGGACCACGACCTCA	CCACCGTAGCACCGATTTTT
OCRL inositol polyphosphate-5-phosphatase (OCRL)	CCTCCCAAGTTGTTTTGGCA	AGGAAGCTGTCTTCTTCCAAGT
inositol polyphosphate 5-phosphatase E (INPP5E)	TCTTGTACAGAAGCCGCCAC	GCCAACGGAATGTTGTCTCG
Synaptojanin 2 (SYNJ2)	CTCTTCAGGAAGAGGCCAAG	ACAGCTGGTTCCAGAAGAAGG

**RT-PCR primers**			
	**Target**	**Forward (5'-3')**	**Reverse primer (5'-3')**
Housekeeping Genes	Beta-Actin	TCACCCACACTGTGCCCATCTACGA	CAGCGGAACCGCTCATTGCCAATGG

Biology Open • Supplementary information

Differentiation Markers	SOX1 (Ectoderm)	GGGAAAACGGGCAAAATAAT	CCATCTGGGCTTCAAGTGTT
Differentiation Markers	Nodal (Mesoderm)	AGACATCATCCGCAGCCTACA	GACCTGGGACAAAGTGACAGTGAA
Differentiation Markers	GATA4 (Endoderm)	TCCAAACCAGAAAACGGAAG	CTGTGCCCGTAGTGAGATGA

**EB Footprint PCR**			
	**Gene name**	**Forward primer (5'-3')**	**Reverse primer (5'-3')**
Plasmid footprint	EBNA-1	ATCGTCAAAGCTGCACACAG	CCCAGGAGTCCCAGTAGTCA

**Junctional PCR**		
	**Forward primer (5'-3')**	**Reverse primer (5'-3')**
Left Junction	CTGTGTCTGAGCCTGCATGTTTG	GGACCTGCCTGGAGAAGGAT
Right Junction	GACCCATGCAGTCCTCCTTAC	CAGGAGGCCTTCCATCTGTTG

**Antibodies used in this study**					
**Primary antibodies**	**Source**	**Dilution**	**Company**	**Catalogue number**	**RRID**
OCRL	Rabbit (polyclonal)	1:200	Thermo Fisher Scientific	PA5-27844	AB_2545320
OCRL (C-2)	Mouse (monoclonal)	1:500	Santacruz	sc-393577	
OCT4	Rabbit	1:100	Thermo Fisher Scientific	A24867	AB_2650999
SOX2	Rat	1:100	Thermo Fisher Scientific	A24759	AB_2651000
SSEA4	Mouse IgG3	1:100	Thermo Fisher Scientific	A24866	AB_ 2651001
Nestin	Mouse (monoclonal)	1:250	Thermo Fisher Scientific	MA1-110	AB_2536821
SOX1	Rabbit (polyclonal)	1:200	Abcam	ab87775	AB_2616563
SOX2	Rabbit (monoclonal)	1:200	Abcam	ab92494	AB_10585428
Ki67	Mouse (monoclonal)	1:200	Abcam	ab8191	AB_306346
PAX6	Mouse (monoclonal)	1:200	Abcam	ab78545	AB_1566562
Musashi 1	Rabbit (monoclonal)	1:200	Abcam	ab52865	AB_881168

Biology Open • Supplementary information

GAPDH	Rabbit (polyclonal)	1:1000	NovusBio	NB100-56875	AB_2107610
mCherry	Rabbit (polyclonal)	1:1000	Thermo Fisher Scientific	PA5-34974	AB_2552323
MAP2	Chicken (polyclonal)	1:1000	Abcam	ab5392	AB_2138153
DCX	Rabbit (polyclonal)	1:200	Abcam	ab18723	AB_732011
Synapsin-1	Rabbit (polyclonal)	1:200	Abcam	ab64581	AB_1281135
**Secondary antibodies**	**Species**		**Company**	**Catalogue number**	**RRID**
Alexa Fluor™ 488	Goat α-Mouse IgG	1:1000	Thermo Fisher Scientific	A11001	AB_2534069
Alexa Fluor™ 488	Goat α-Rabbit IgG	1:1000	Thermo Fisher Scientific	A11034	AB_2576217
Alexa Fluor™ 568	Donkey α-Rabbit IgG	1:1000	Thermo Fisher Scientific	A10042	AB_2534017
Alexa Fluor™ 568	Goat α-Mouse IgG	1:1000	Thermo Fisher Scientific	A11004	AB_2534072
Alexa Fluor™ 633	Goat α-Rabbit IgG	1:1000	Thermo Fisher Scientific	A21070	AB_2535731
Alexa Fluor™ 594	Donkey α-Rabbit	1:250	Thermo Fisher Scientific	A24870	Not available
Alexa Fluor™ 488	Donkey α-Rat	1:250	Thermo Fisher Scientific	A24876	AB_2651007
Alexa Fluor™ 488	Goat α- Mouse IgG3	1:250	Thermo Fisher Scientific	A24877	AB_2651008
Alexa Fluor™ 568	Goat α-Chicken IgG	1:1000	Thermo Fisher Scientific	A11041	AB_2534098
